# Association between erythrocyte membrane fatty acids and gut bacteria in obesity-related cognitive dysfunction

**DOI:** 10.1186/s13568-023-01655-3

**Published:** 2023-12-20

**Authors:** Tong Zhao, Hongying Huang, Jinchen Li, Jingyi Shen, Cui Zhou, Rong Xiao, Weiwei Ma

**Affiliations:** School of Public Health, Beijing Key Laboratory of Environmental Toxicology, Capital Medical University, Beijing, 100069 People’s Republic of China

**Keywords:** Obesity, Intestinal microbes, Mild cognitive impairment, Fatty acids

## Abstract

Obesity increases the risk of cognitive impairment and dementia, and the gut microbiota can affect brain cognitive function and obesity through a variety of pathways such as the gut-brain axis. This study aimed to discover how fatty acid affect cognitive function by regulating intestinal flora in obesity. Obese subjects were recruited for cognitive function assessment, and participants were divided into obese group with cognitive impairment (MCI, n = 49) and obese cognitively normal group (Non_MCI, n = 55). In the erythrocyte membrane, the proportion of polyunsaturated fatty acids (PUFA), linoleic acid (C18:2 n-6) and arachidonic acid (C20:4 n-6) and n-6/n-3 ratio was higher in the MCI group than in the Non_MCI group. However, the α-linolenic acid (C18:3 n-3) percentage of the erythrocyte membrane was lower in the MCI group. We found that *Coriobacteriales_Incertae_Sedis* was positively correlated with erythrocyte membrane C20:4 n-6 and n-6 PUFA and negatively correlated with cognitive scores in obese patients. In addition, several of the functional pathways we predicted were significantly different in the MCI and Non_MCI groups. Higher levels of n-6/n-3 polyunsaturated fatty acids ratio in the erythrocyte membranes may influence the inflammatory response in the organism causing obesity induced cognitive damage. Moreover, high levels of n-6/n-3 polyunsaturated fatty acids ratio may also affect the intestinal flora of obese patients, which in turn may affect the cognitive function of obese patients.

**Supplementary Information:**

The online version contains supplementary material available at 10.1186/s13568-023-01655-3.

## Key Points

1. Obesity with or without cognitive impairment differs in gut flora.

2. Alterations in erythrocyte membrane fatty acids in obesity with cognitive impairment.

3. Relationship between intestinal bacteria and erythrocyte membrane fatty acids.

**Supplementary Information:**

The online version contains supplementary material available at 10.1186/s13568-023-01655-3.

## Introduction

According to the World Health Organization (WHO), the prevalence of overweight and obesity has increased dramatically around the world and has become one of the serious public health events (Ma & Xiao 2021). As one of the main symptoms of metabolic syndrome, obesity is closely related to many diseases, such as type 2 diabetes mellitus (T2DM), hyperlipidemia, hypertension, coronary heart disease, stroke, tumors, neurodegenerative disease, etc. (Kivipelto et al. 2018). Among many adverse health outcomes, it is strongly associated with the subsequent occurrence of cognitive impairment and is a risk factor for dementia and Alzheimer’s disease (AD) (Whitmer & Gunderson 2007). Its association with cognitive dysfunction can be attributed to the metabolic consequences of visceral obesity (Saklayen 2018).

An unhealthy diet is an important factor in obesity. Epidemiological studies suggest an association between obesity due to dietary fat intake and increased risk of cognitive impairment (Rizzi et al. 2014). Animal experiments show that feeding an n-6 polyunsaturated fatty acids (n-6 PUFA-rich) diet may adversely affect cognitive function in obese mice by modulating inflammatory markers and inflammatory signaling pathways in the brain and intestine of mice (Fan et al. 2022). n-3 polyunsaturated fatty acids (n-3 PUFA) is an essential dietary nutrient that plays a central role in learning, memory, and regulation of the nervous and cardiovascular systems (Su 2010). A former study has shown that n-3 PUFA supplementation can reduce plasma c-reactive protein (CRP) and systemic inflammatory response, and improve cerebrovascular function (Kuszewski et al. 2020). A cross-sectional study including 299 women from Australia showed that women with lower levels of n-3 PUFA in erythrocyte membranes had decreased cognitive performance in the attention domain (Cook et al. 2019). Our earlier study also showed that an increase in n-3 PUFA in erythrocyte membranes was accompanied by a rise in cognitive performance in the obese population, which may be a protective factor for cognitive function in the obese population. In contrast, higher intake of n-6 PUFA may exacerbate the cognitive decline in obese individuals (Shen et al. 2022). The effect of dietary fatty acids on the cognition of obese patients is most likely due to the triggering of systemic and neuroinflammatory disorders.

It has been shown that excessive or insufficient intake of fatty acids also affects the human intestinal flora leading to ecological disorders (Tsutsumi et al. 2021), which in turn could affect cognitive function. Mice fed n-3 PUFA-containing diet were found to have increased abundance of *Bifidobacterium* and *Lactobacillus* in their feces and healthy hypothalamic-pituitary-adrenal axis activity. This result suggests that dietary interventions, particularly n-3 PUFA, may have an impact on behavioral outcomes and may be mediated by the gut-brain axis (GBA) (Mazahery et al. 2019). Higher intake of n-6 PUFA is associated with reduced *Bifidobacterium* numbers and some immune functions, such as antigen expression and pro-inflammatory cytokines (Robertson et al. 2017). Pusceddu’s team demonstrated that long-term exposure to a diet with a low n-6/n-3 ratio benefited anxiety and cognition in non-stressed female rats and partially restored the disturbed gut microbiota composition in female rats (Pusceddu et al. 2015).

Some microRNAs (miRNAs) act as important regulators of various biological functions in the brain, including synaptic plasticity and neurogenesis, and may indirectly affect neurogenesis by regulating neural stem cell proliferation and repair (Grasso et al. 2014). Studies on middle-aged and older Chinese people with cognitive impairment have shown that certain microorganisms in the gut such as *Proteobacteria* and *Gammaproteobacteria* are associated with miR-107 and miR-186 to affect cognitive function in humans (Zhang et al. 2021). Moreover, studies have shown that supplementation with the proper ratio of n-6/n-3 PUFA can selectively enhance miRNA clusters such as miR-30, miR-103 associated with AD (Chakraborty et al. 2017).

Therefore, in this study, we aimed to investigate the changes in erythrocyte membrane fatty acids, miRNAs, and intestinal flora between mild cognitive impairment (MCI) group and cognitively normal (Non_MCI) group and to find some associations from them. In order to provide a theoretical basis for the prevention and treatment of the development of cognitive impairment in obese patients.

## Materials and methods

### Participants and groups

The study recruited volunteers in Daxing District, Beijing. Subjects were selected between 45 and 75 years of age, regardless of gender. People who had taken antibiotics and probiotics within 3 months were excluded; people with acute or chronic gastrointestinal disease were excluded; people with a history of cancer disease, head trauma, encephalitis, and other central nervous diseases, or any type of definite mental illness were excluded; people with reading, hearing, or vision impairments were excluded; people who had been treated for a disease were excluded; or people who were losing the ability to care for themselves were excluded. The height and weight of the recruited volunteers were measured uniformly by professionally trained investigators and 105 subjects were diagnosed as obese with a body mass index (BMI) ≥ 28.0 kg/m^2^. According to the diagnostic criteria for MCI (Pan et al. 2020), (1) years of education ≤ 6 years, Montreal Cognitive Assessment Scale (MoCA) ≤ 19; (2) 7 < years of education ≤ 12 years, MoCA ≤ 22; (3) years of education > 12 years, MoCA ≤ 24. Subjects with missing educational information and MoCA scores were excluded. The remaining 104 obese subjects were divided into an obese with cognitive impairment group (MCI, n = 49) and an obese but cognitively normal group (Non_MCI, n = 55).

The population study was approved by the ethics committee of Capital Medical University (Ethics Review Number: Z2019SY011). Subjects voluntarily signed an informed consent form and were surveyed. Subjects had the right to learn about, choose and withdraw from the program.

### Baseline data collection

A face-to-face questionnaire survey were used to gather data on sociodemographic factors. Sociodemographic factors such as name, age, gender, smoking, alcohol consumption, exercise and history of hypertension were collected in this study. As in our earlier study, we used the “Clinical Guidelines for the Prevention and Treatment of Type 2 Diabetes in the older people in China” published in 2022 that states that diabetes mellitus is diagnosed by fasting blood glucose (fasting blood glucose ≥ 7.0 mmol/L). According to the “Chinese guidelines for the prevention and treatment of dyslipidemia in adults (2016 revision)”, it was stated that serum total cholesterol (TC) ≥ 6.2 mmol/L; serum triglyceride (TG) ≥ 2.3 mmol/L; serum TC ≥ 6.2 mmol/L and serum TG ≥ 2.3mmol/L; serum high-density lipoprotein cholesterol (HDL-C)  < 1.0 mmol/L. Hyperlipidemia could be diagnosed if any of the above conditions were met.

### Physical examination

As was mentioned in our previous study (Shen et al. 2022), briefly, measurements of height (m), weight (kg), waist circumference (cm) and hip circumference (cm) were taken. BMI and waist-hip ratios (WHR) were calculated according to the following formula: BMI = weight (kg)/ height (m)^2^; WHR = waist circumference (cm)/ hip circumference (cm).

#### Cognitive function examination

Overall cognition was assessed by using the Minimum Mental State Examination (MMSE) and the MoCA, and the participants’ scale score information and each visit record and clinical data were stored in a computer (Ding et al. 2018).

The MMSE scale has high specificity and sensitivity for the diagnosis of dementia, and is relatively simple in the assessment of executive function, so the sensitivity for MCI assessment is relatively poor. The sensitivity of MoCA for the detection of MCI is significantly better than that of MMSE. This study included MCI-sensitive MoCA scales, which are now commonly used. The MMSE scale includes orientation (up to 10 points), memory (up to 3 points), attention (up to 5 points), delayed recall (up to 3 points), language (up to 9 points); The total score of the MMSE is 30 points. The MoCA scale includes MoCA naming ability (up to 3 points), MoCA orientation (up to 6 points), MoCA delayed recall (up to 5 points), MoCA abstract thinking (up to 2 points), MoCA language skills (up to 3 points), MoCA visuospatial ability (up to 5 points), and MoCA attention (up to 6 points) for a total score of 30.

In this study, the MoCA score was mainly used as the basis for determining mild cognitive impairment, while the MMSE score was only used as a reference for the subjects’ cognitive status.

### Blood sample collection and detection

Blood samples were collected using the anterior elbow vein of each participant. Enzyme colorimetric method was used to determine TG, TC, HDL-C, and low-density lipoprotein cholesterol (LDL-C) in serum (Cobas C501, Randox, Northern Ireland, UK). Fasting blood glucose levels were determined by hexokinase (Cobas C501, Randox, Northern Ireland, UK). Apolipoprotein E (ApoE) was determined by immunoturbidity method (Cobas C501, Randox, Northern Ireland, UK) (Fan et al. 2021). Enzyme linked immunosorbent assay (ELISA) was adopted to detect the levels of interleukin-3 (IL-3), IL-12, interferon-γ (IFN-γ) (Beijing Meichen Lianchuang Biotechnology Co., Ltd, Beijing, China).

### RNA purification and miRNA analysis

Total RNA extracted from plasma was purified by spin column method using the miRNeasy Serum/Plasma Kit (Qiagen, Hilden, Germany) according to the manufacturer’s protocol (Liu et al. 2021b). Total RNA in blood cells was extracted by a spin column method using the miRNeasy Kit (Qiagen, Hilden, Germany). miRNAs were reverse transcribed into cDNA using the QuantiNova Reverse Transcription Kit (Qiagen, Hilden, Germany), followed by the real-time RT-PCR according to the manufacturer’s protocol.

### Analysis of fatty acids in erythrocyte membranes

Blood samples were collected using a tube with ethylene diamine tetraacetic acid (EDTA), plasma was separated and normal saline was added to isolate red blood cells. Then, the pre-chilled 10 mmol/L pH 7.4 Tris-HCI buffer solution was added to the mixture at a ratio of 1:40 and placed in a 4 °C freezer for 2 h to complete hemolysis. Then, the mixture was centrifuged at 4 °C to pellet the erythrocyte. The supernatant was discarded and washed for 3 times. Finally, Erythrocyte membrane sample was obtained. Gas chromatography analysis was then employed to determine the fatty acid composition in erythrocyte membrane according to the protocol in our previous study (Amirkalali et al. 2018).

The omega-3 index (O3I), defined as the sum of eicosapentaenoic acid (EPA) and docosahexaenoic acid (DHA) expressed as a percentage of total erythrocyte membrane fatty acids, is a reliable measure of dietary n-3 PUFA intake and reflects long-term n-3 PUFA status (Cook et al. 2019).

### Stool sample collection and 16 S rRNA sequencing

About 2 g of fresh, naturally eliminated feces were collected from subjects on an empty stomach, loaded into test tubes, and stored at a low temperature of -80 °C.

Genomic DNA was extracted from the subject’s stool, then PCR amplification was performed according to the 338F_806R region, and the PCR products were quantified by QuantiFluor^TM^-ST Blue Fluorescence Quantification System (Promega, America), mixed in the appropriate ratio according to the requirement of sequencing volume for each sample, followed by Miseq library construction as well as sequencing. The DNA fragments were used as templates and the fixed base sequences on the chip were used as primers for PCR synthesis, and the target DNA fragments to be tested were synthesized on the chip to generate DNA clusters, and then the reaction plate surface was scanned with a laser to read the nucleotide species polymerized up by the first reaction of each template sequence, and the results of the collected fluorescence signals were counted to obtain the sequences of the template DNA fragments.

### Bioinformatics analysis

Diversity data analysis of all samples in the MCI and non-MCI groups was completed, and a total of 15,517,560 optimized sequences were obtained, with an average sequence length of 411 bp. All sequences were divided into OTU based on 97% similarity level and subsequently analyzed for bioinformatic statistics.

Based on the results of OTU and its clustering analysis and the information analyzed, the structural composition of the intestinal flora can be analyzed at the level of each genus. In this study, we mainly performed Venn diagram analysis, dilution curve analysis, alpha and beta diversity analysis, Partial least squares Discriminant Analysis (PLS-DA) analysis, LEfSe analysis and Tax4Fun functional prediction analysis on intestinal flora. The above data analysis results such as alpha index and OTU table are based on the Meiji Biotech cloud platform (https://www.majorbio.com/web/login/passport/login-email). (See supplement material for details)

### Accession number

The sequences have been deposited in the NCBI Sequence Read Archive database under the bioproject number: PRJNA978383.

### Statistical analysis

SPSS v26.0 software (https://www.ibm.com/products/spss-statistics) was used in this study, and all data were tested for normality. *t*-test was used for data that met normality, and nonparametric rank sum test was used for data that did not meet normality. The Chi-squared test was used for unordered categorical variables, Fisher’s exact probability test was used if the statistical results did not meet the conditions of the χ^2^ test, and the nonparametric rank sum test was used for ordered categorical variables. The Mann-Whitney U (M-W U) rank sum test was used to analyze the species differences between the two groups. Post-hoc test Scheffe method was used to analyze the species differences between the two groups. The measurement data were expressed as mean ± standard deviation (mean and SD), and the count data were reported as n (%). All the above statistical results *P* < 0.05 indicates that the difference was statistically significant.

## Results

### Basic information

The basic information of the obese individuals with MCI and Non_MCI were statistically tested. No differences were found between the two groups in gender, ethnicity, age, height, weight, residence, smoking and drinking, exercise, disease history, etc. after statistical testing (Table 1).

**Table 1:** Basic information about the subject

Variable	MCI (n = 49)	Non_MCI (n = 55)	χ^2^	*P*
Gender			1.157	0.282
male	13(26.5%)	20(36.4%)		
female	36(73.5%)	35(63.6%)		
Age	60.040 ± 6.461	58.560 ± 7.686		0.178
BMI (kg/m^2^)	30.833 ± 2.493	30.618 ± 2.600		0.360
Waist-hip ratio	0.918 ± 0.059	0.912 ± 0.065		0.257
Educational				0.854
illiterate	4(8.2%)	1(1.8%)		
elementary school	10(20.4%)	11(20.0%)		
junior high school	21(42.9%)	33(60.0%)		
high school	11(22.4%)	9(16.4%)		
secondary and junior college	3(6.1%)	1(1.8%)		
Degree of physical exertion				0.053
mild	38(77.5%)	33(60.0%)		
moderate	9(18.4%)	17(30.9%)		
severe	2(4.1%)	5(9.1%)		
Smoking			3.110	0.160
never	44(89.8%)	45(81.8%)		
smoking for 6 consecutive months	4(8.2%)	10(18.2%)		
smoking for 6 months but not continuously	1(2.0%)	0(0.0%)		
Drinking			0.151	1.000
never	32(68.1%)	38(69.1%)		
former	3(6.4%)	3(5.5%)		
current	12(25.5%)	14(25.4%)		
Exercise			1.796	0.180
no	10(20.4%)	6(10.9%)		
yes	39(79.6%)	49(89.1%)		
History of hypertension			0.958	0.917
No	22(44.9%)	26(47.3%)		
yes	27(55.1%)	28(50.9%)		
unknown	0(0.0%)	1(1.8%)		
Diabetes testing			0.006	0.938
no	38(77.6%)	43(78.2%)		
yes	11(22.4%)	12(21.8%)		
Hyperlipidemia testing			2.792	0.095
No	27(55.1%)	39(70.9%)		
yes	22(44.9%)	16(29.1%)		

The mean and standard deviation (SD) were used to express the measurement data, and n (%) was used to express the count data. MCI: obesity with cognitive impairment group; Non_MCI: obese but cognitively normal group, *P < 0.05

### The comparison of fatty acid composition in erythrocyte membrane

To assess differences in fatty acid intake, gas chromatography is performed to determine the fatty acid composition of the erythrocyte membrane. The results showed higher proportions of methyl pentadecanoate (C15:0), linoleic acid (C18:2 n-6), arachidonic acid (C20:4 n-6, AA), n-6 PUFA, and n-6/n-3 PUFA in the MCI group as compared to the non-MCI group. In contrast, C20:3 n-3 levels in erythrocyte membranes was lower than in the Non-MCI group. In the MCI group, the proportion of lignoceric acid (C24:0) in erythrocyte membrane saturated fatty acids was lower than that in the Non_MCI group, while the proportion of C15:0 was higher than that in the Non_MCI group. The proportion of nervonic acid (C24:1) in erythrocyte monounsaturated fatty acids was lower in the MCI group compared to the Non_MCI group. The proportion of α-linolenic acid (C18:3 n-3, ALA) in erythrocyte membrane in the MCI group was lower than that in the Non_MCI group (Table 2).

**Table 2:** Comparison of fatty acid composition of erythrocyte membranes between the two groups

Variable	MCI (n = 49)	Non_MCI (n = 55)	*P*
C10:0%	0.022 ± 0.154	0.000 ± 0.000	0.294
C11:0%	0.880 ± 4.338	0.041 ± 0.302	0.261
C12:0%	0.264 ± 0.823	0.426 ± 0.792	0.164
C13:0%	0.051 ± 0.305	0.365 ± 1.173	0.212
C14:0%	0.183 ± 0.348	0.498 ± 0.884	0.490
C15:0%	0.982 ± 1.595	0.836 ± 2.251	0.028*
C16:0%	27.995 ± 7.838	30.322 ± 5.245	0.112
C17:0%	0.101 ± 0.160	0.053 ± 0.105	0.101
C18:0%	19.019 ± 6.492	20.331 ± 4.834	0.119
C20:0%	0.000 ± 0.000	0.020 ± 0.086	0.096
C21:0%	0.007 ± 0.052	0.000 ± 0.000	0.294
C23:0%	0.025 ± 0.129	0.000 ± 0.000	0.136
C24:0%	0.000 ± 0.000	0.042 ± 0.144	0.017*
SFA%	49.529 ± 9.672	52.935 ± 9.891	0.082
C14:1%	0.025 ± 0.123	0.031 ± 0.231	0.518
C15:1%	0.028 ± 0.194	0.059 ± 0.311	0.618
C16:1%	0.073 ± 0.149	0.070 ± 0.180	0.574
C17:1%	0.004 ± 0.026	0.010 ± 0.041	0.364
C18:1 n-9%	10.769 ± 2.099	10.292 ± 3.855	0.059
C20:1%	0.041 ± 0.180	0.088 ± 0.224	0.107
C24:1%	0.520 ± 1.048	1.787 ± 2.760	0.011*
MUFA%	11.459 ± 2.398	12.337 ± 4.452	0.398
C18:2 n-6%	13.329 ± 2.889	11.939 ± 3.284	0.014*
C18:3 n-6%	0.014 ± 0.095	0.027 ± 0.156	0.618
C20:3 n-6%	1.170 ± 0.700	0.998 ± 0.647	0.173
C20:4 n-6%	19.413 ± 5.407	16.699 ± 5.148	0.009*
n-6 PUFA%	33.925 ± 7.683	29.663 ± 7.506	0.009*
C18:3 n-3%	0.000 ± 0.000	0.094 ± 0.418	0.030*
C20:3 n-3%	0.437 ± 3.059	0.478 ± 3.513	0.956
C20:5 n-3%	0.000 ± 0.000	0.005 ± 0.035	0.341
C22:6 n-3%	4.614 ± 1.297	4.435 ± 1.505	0.245
n-3 PUFA%	5.051 ± 3.703	5.012 ± 3.971	0.348
C20:2%	0.035 ± 0.108	0.053 ± 0.127	0.375
PUFA%	39.011 ± 8.037	34.728 ± 8.166	0.023*
n-6/n-3%	7.682 ± 1.983	6.812 ± 1.796	0.020*
O3I%	0.046 ± 0.013	0.044 ± 0.015	0.249

The data is represented by mean and SD. MCI: obesity with cognitive impairment group; Non_MCI: obese but cognitively normal group. SFA: Saturated fatty acids; MUFA: Monounsaturated fatty acids; PUFA: Polyunsaturated fatty acids; TFA: Trans fatty acids; n-6: omega-6 fatty acids; n-3: omega-3 fatty acids; O3I: omega-3 index; *P < 0.05

### Analysis of serum lipid metabolism indexes and inflammatory factors in both groups

We further explored the levels of blood biochemical parameters in the serum of both groups of subjects and found that the serum levels of HDL-C were lower and the levels of IL-3 and IL-12 were higher in the MCI group compared to the Non_MCI group (Table 3).

**Table 3:** Two groups studied blood biochemical parameters of the population

Variable	MCI (n = 49)	Non_MCI (n = 55)	*P*
TC (mmol/L)	5.298 ± 0.987	5.148 ± 1.095	0.415
TG (mmol/L)	1.900 ± 1.099	1.637 ± 1.014	0.164
HDL-C (mmol/L)	1.320 ± 0.311	1.405 ± 0.295	0.055
LDL-C (mmol/L)	3.273 ± 0.745	3.177 ± 0.891	0.486
FBG (mmol/L)	6.134 ± 1.855	6.289 ± 2.355	0.866
FFA (mmol/L)	0.853 ± 0.458	0.986 ± 0.531	0.250
ApoE (mg/L)	54.177 ± 14.984	53.235 ± 17.396	0.716
TLR1 (ng/ml)	4.715 ± 0.944	4.854 ± 0.769	0.386
TLR2 (ng/ml)	24.656 ± 10.920	21.968 ± 9.799	0.185
TLR4 (ng/ml)	7.401 ± 1.197	7.366 ± 1.350	0.663
IL-3 (pg/ml)	73.884 ± 35.160	69.457 ± 32.616	0.561
IL-12 (pg/ml)	54.254 ± 25.321	44.991 ± 21.116	0.059
IFN-γ (pg/ml)	871.252 ± 325.819	816.120 ± 261.140	0.337

The data is represented by mean and SD. MCI: obesity with cognitive impairment group; Non_MCI: obese but cognitively normal group. TC: total cholesterol; TG: total triglycerides; HDL-C: high-density lipoprotein cholesterol; LDL-C: LDL cholesterol; FBG: fasting blood glucose; FFA: free fatty acids; ApoE: apolipoprotein E; TLR1: toll-like receptor 1; TLR2: toll-like receptor 2; TLR4: toll-like receptor 4; IL-3: interleukin-3; IL-12: interleukin 12; IFN-γ: interferon gamma; *P < 0.05

We then correlated erythrocyte membrane fatty acids and plasma cytokine levels in the subjects. The results revealed that n-6 PUFA, LA and AA were positively correlated with IL-3, toll-like receptor 2 (TLR2) and TLR4. ALA was positively correlated with low-density lipoprotein cholesterol (LDL-C) (Supplementary Fig S1).

### Analysis of plasma microRNA in both groups

**Fig. 1:**
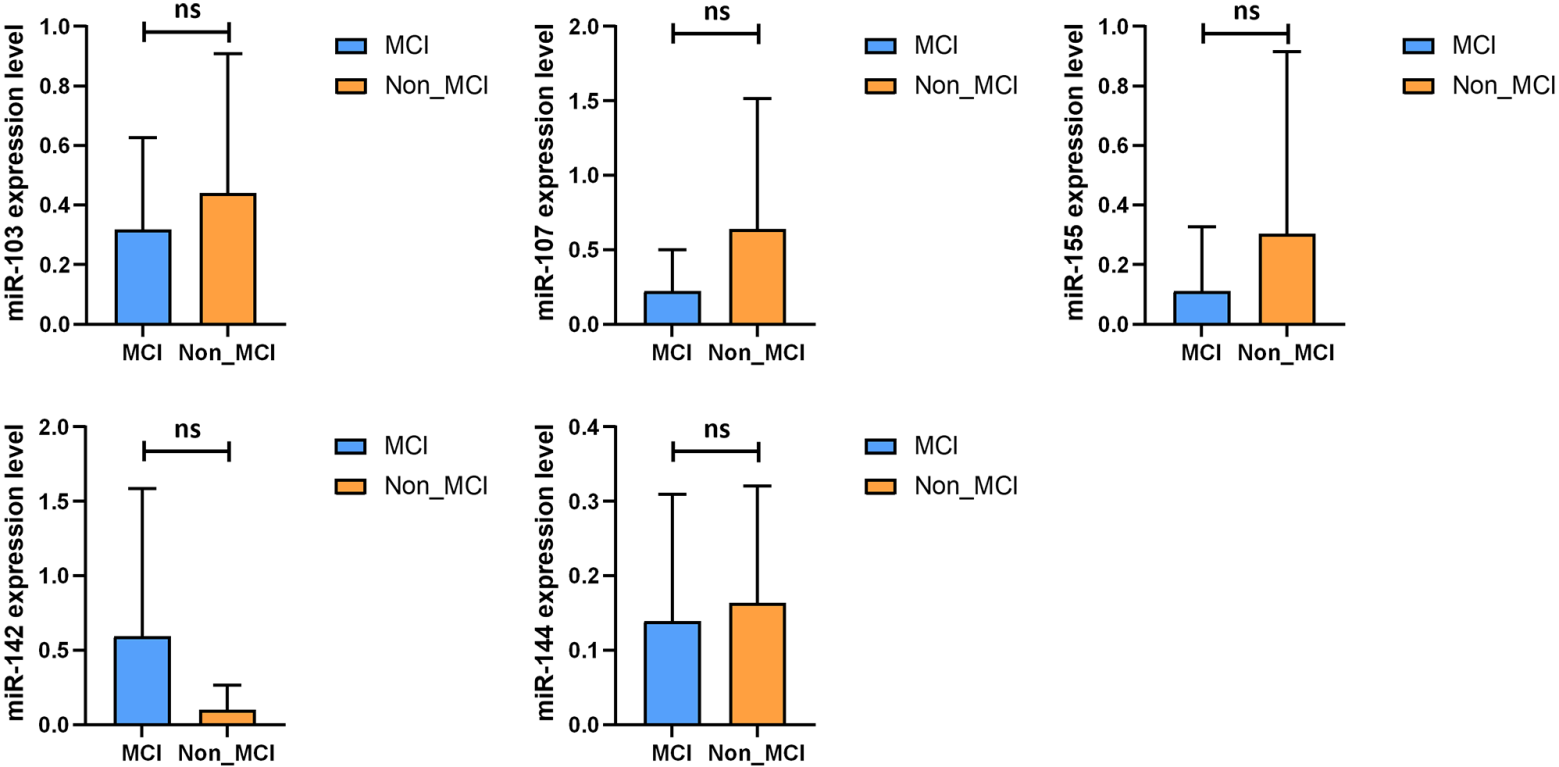
Comparison of plasma miRNA differences in the MCI and Non_MCI groups. We removed the abnormal values in the MicroRNA (miRNA) data of the two populations and replaced them with the mean values of each group. MCI: obesity with cognitive impairment group; Non_MCI: obese but cognitively normal group; *P < 0.05

The comparison of miRNA in plasma between the two groups then revealed that miR-103, miR-107, miR-144 and miR-155 were decreased in the MCI group, while miR-142 were upregulated in the MCI group (Fig. 1). These results suggest that miRNA levels were altered in the blood of patients with obesity with mild cognitive impairment.

### Basic information of two groups of intestinal flora

Venn diagrams were drawn based on OTU clustering. A total of 1100 OTUs were generated by 104 samples in the two groups, of which 799 OTUs were in the two groups, and 177 OTUs in the obese group with MCI and 124 in the obese cognitively normal group, respectively (Supplementary Fig S2a). These results suggest that the cognitively impaired and cognitively normal groups were mostly identical in terms of gut bacterial composition, but a small portion was still specific.

The dilution curve was close to flattening and the coverage of Good’s coverage reaches 99.97%, which together indicates that the detection rate of intestinal flora in the feces of the two groups of obese people was close to saturation, and the current sequencing volume could cover most of the species in the sample (Supplementary Fig S2b). It was shown that the obtained data, the depth of sequencing of the gut flora and the sample size were reliable. The depth of sequencing could cover all species in both sets of fecal samples.

### Alpha diversity analysis

The six alpha diversity indices, Ace, Chao, Shannon, Simpson, Sobs, and Coverage were used to explore the richness, diversity and coverage of intestinal flora in the two groups of obese people. Statistical tests showed no significant differences in the richness, diversity and coverage of intestinal flora in feces between the two groups of obese people, which reflects that there was no significant difference in alpha diversity of the gut microbiota between the MCI and Non_MCI groups (Table 4).

**Table 4:** Alpha diversity index analysis

Variable	MCI (n = 49)	Non_MCI (n = 55)	*P*
Sobs	209.370 ± 66.985	195.360 ± 71.938	0.309
Shannon	3.291 ± 0.546	3.129 ± 0.654	0.175
Simpson	0.089 ± 0.059	0.110 ± 0.091	0.334
Ace	262.964 ± 74.356	256.443 ± 87.026	0.684
Chao	258.159 ± 81.313	247.977 ± 90.131	0.548
coverage	0.998 ± 0.001	0.998 ± 0.001	0.998

The data is represented by mean and SD. Ace and Chao indices reflect community richness, Shannon and Simpson indices reflect community diversity, Coverage reflects the coverage of the community; MCI: obesity with cognitive impairment group; Non_MCI: obese but cognitively normal group; *P < 0.05

### Structural changes in the gut microbial communities

Based on two different Beta diversity distance algorithms (Unweighted UniFrac and Weighted UniFrac), the ANOSIM intergroup difference test was used to analyze the overall structure of the intestinal flora of the two groups.

**Fig. 2:**
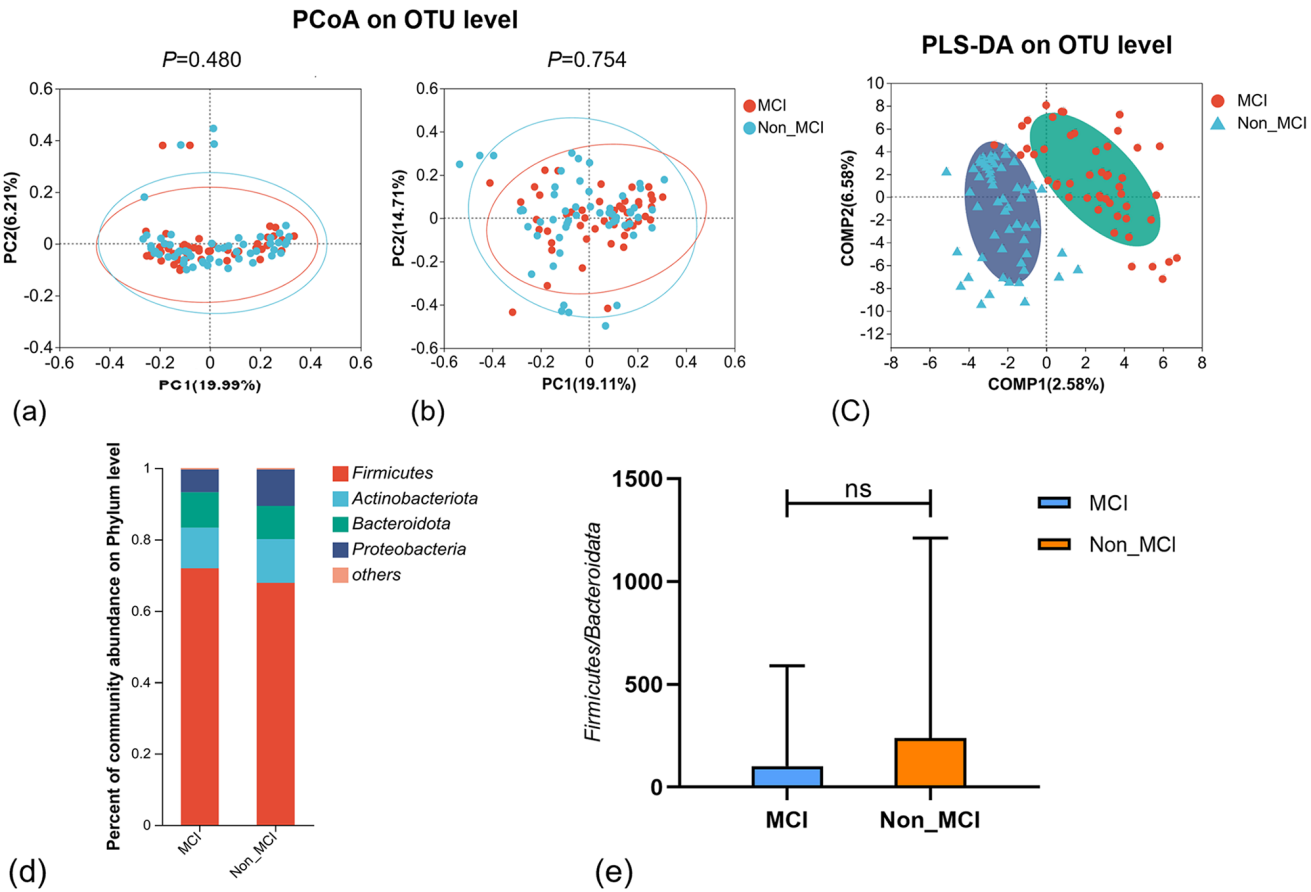
Structural changes in the gut microbial communities. PCoA analysis (principal co-ordinates analysis), or principal coordinate analysis, is a non-binding data dimensionality reduction analysis method that can be used to study the similarity or difference in the composition of sample communities. (**a**) PCoA analysis based on Unweighted UniFrac distance algorithm; (**b**) PCoA analysis based on Weighted UniFrac distance algorithm; (**c**) PLS-DA analysis, This analysis method needs to group the tested samples according to categories, distinguish each group during calculation, ignore the random differences in the group, and have better sample differentiation performance when the observation factor is much larger than the total sample size. (**d**) Community analysis sunburst plot on phylum level: MCI and Non_MCI; (**e**) The Mann-Whitney U (M-W U) test was used to statistically test the *Firmicutes/Bacteroidota* ratio; MCI: obesity with cognitive impairment group; Non_MCI: obese but cognitively normal group; *P < 0.05

Principal co-ordinates analysis (PCoA) plot according to unweighted UniFrac distances algorithm (similarity analysis, R=-0.0015; *P* = 0.480); The principal component (PC) scores were PC1 = 19.99% and PC2 = 6.21%, respectively (Fig. 2a). PCoA plot according to weighted UniFrac distances algorithm (similarity analysis, R=-0.0096; *P* = 0.754); The principal component (PC) scores were PC1 = 19.11% and PC2 = 14.71%, respectively (Fig. 2b). These results showed that there was no significant difference in the composition of intestinal microbial communities between the two groups (*P* > 0.05).

The PLS-DA results showed that the two groups of MCI and Non-MCI samples could be clearly distinguished and clustered into two taxa, indicating significant differences in the composition of the fecal flora between MCI and Non_MCI. In addition, significant differences in the composition of the intestinal flora were illustrated by the dispersion of the sample point distribution compared between the MCI and Non-MCI groups (Fig. 2c).

The dominant phylum of the MCI and Non_MCI group are *Firmicutes, Actinobacteriota, Bacteroidota*, and *Proteobacteria*. Mann-Whitney U tests were performed on the *Firmicutes/Bacteroidota* ratio in the intestines of the MCI and Non_MCI groups, and no significant difference in the *Firmicutes/Bacteroidota* ratio between the two groups (*P* = 0.521) (Fig. 2e). However, *Actinobacteriota* and *Proteobacteria* showed a gradual upward trend between the MCI group and the Non_MCI group, and *Actinobacteriota* accounted for 11.40% and 12.40% (Fig. 2d), respectively between the two groups. *Proteobacteria* accounted for 6.39% and 10.20% respectively between the two groups (Fig. 2d).

### Taxonomy-based comparisons of the intestinal microbiota

According to the community abundance information of fecal intestinal flora in the two groups, the statistical method of Mann-Whitney U (M-W U) rank sum test was used to test the differences between groups at the order, family, genus and species levels of the stool samples of the MCI group and the Non_MCI group.

**Fig. 3:**
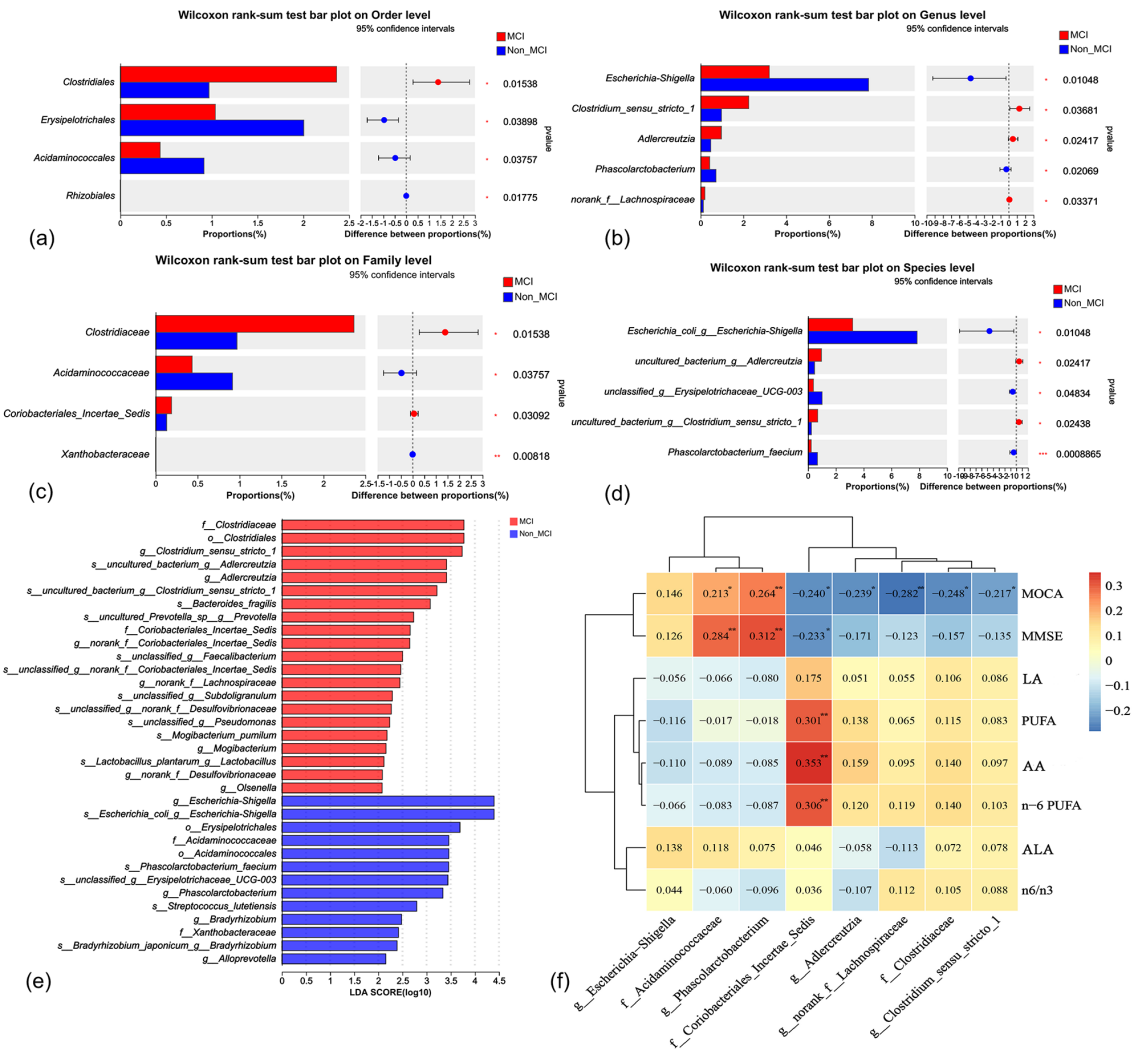
Taxonomy-based comparisons of the intestinal microbiota. Compare the relative abundance of the first five major bacterial orders, families, genera and species. (**a**) Comparison of order level, MCI and Non_MCI group dominance purposes; (**b**) Comparison of family level, MCI and Non_MCI group dominance purposes; (**c**) Comparison of genus level, MCI and Non_MCI group dominance purposes; (**d**) Comparison of species level, MCI and Non_MCI group dominance purposes; (**e**) LEfSe uses linear discriminant analysis (LDA) to estimate the magnitude of the effect of each component (species) abundance on the differential effect. (**f**) The correlation heatmap shows the association of two groups of gut microbiota with their cognitive scores and erythrocyte membrane fatty acids. MCI: obesity with cognitive impairment group; Non_MCI: obese but cognitively normal group; MoCA: *P < 0.05, **P < 0.01, ***P < 0.001

The results showed that at the order level (Fig. 3a), the relative abundance of *Erysipelotrichales, Clostridiales, Acidaminococcales* and *Rhizobiales* was significantly different in the feces of the two groups, and the relative abundance of *Erysipelotrichales* and *Acidaminococcales* was higher in the Non_MCI group. On the other hand, *Clostridiales* were less abundant. At the family level (Fig. 3c), the relative abundances of *Xanthobacteraceae*, *Coriobacteriales_Incertae_Sedis, Clostridiaceae* and *Acidaminococcaceae* were significantly different, and the abundances of *Coriobacteriales_Incertae_Sedis* and *Clostridiaceae* were higher in the MCI group. At the genus level (Fig. 3b), the relative abundance of *Escherichia-Shigella, Clostridium_sensu_stricto_1, Adlercreutzia*, and *Phascolarctobacterium* differed significantly. At the species level (Fig. 3d), the relative abundance of *Escherichia_coli_g_Escherichia-Shigella, unclassified_g_Erysipelotrichaceae_UGG-003, uncultured_bacterium_g_Adlercreutzia*, and *Phascolarctobacterium_faecium* was significantly different.

Linear discriminant analysis Effect Size (LEfSe) analysis (Linear Discriminant Analysis: LDA threshold of 2) found that between the MCI group and the Non_MCI group, the fecal intestinal flora of the MCI group was significantly enriched in *Clostridiaceae, Clostridiales, Clostridium_sensu_stricto_1, Adlercreutzia, Coriobacteriales_Incertae_Sedis*, and *norank_f_Lachnospiraceae*. The Non_MCI group was significantly enriched *Escherichia-Shigella, Erysipelotrichales, Phascolarctobacterium, Xanthobacteraceae*, and *Alloprevotella* (Fig. 3e). This is generally consistent with the results of the Mann-Whitney U test.

We used Spearman’s correlation analysis to analyze the correlation between the two groups differing at the family and genus levels for intestinal flora and cognitive scores and erythrocyte membrane fatty acids, and to plot Heatmap.

The correlation heatmap results showed that *Adlercreutzia, norank_f_Lachnospiraceae, Clostridiaceae*, and *Clostridium_sensu_stricto_1* were negatively correlated with cognitive scores, while *Acidaminococcaceae* and *phascolarctobacterium* were positively correlated with cognitive scores. We also found that *Coriobacteriales_Incertae_Sedis* was negatively correlated with cognitive scores and positively correlated with Arachidonic acid (AA), n-6 PUFA in erythrocyte membranes (Fig. 3f).

#### Tax4Fun function prediction

**Fig. 4:**
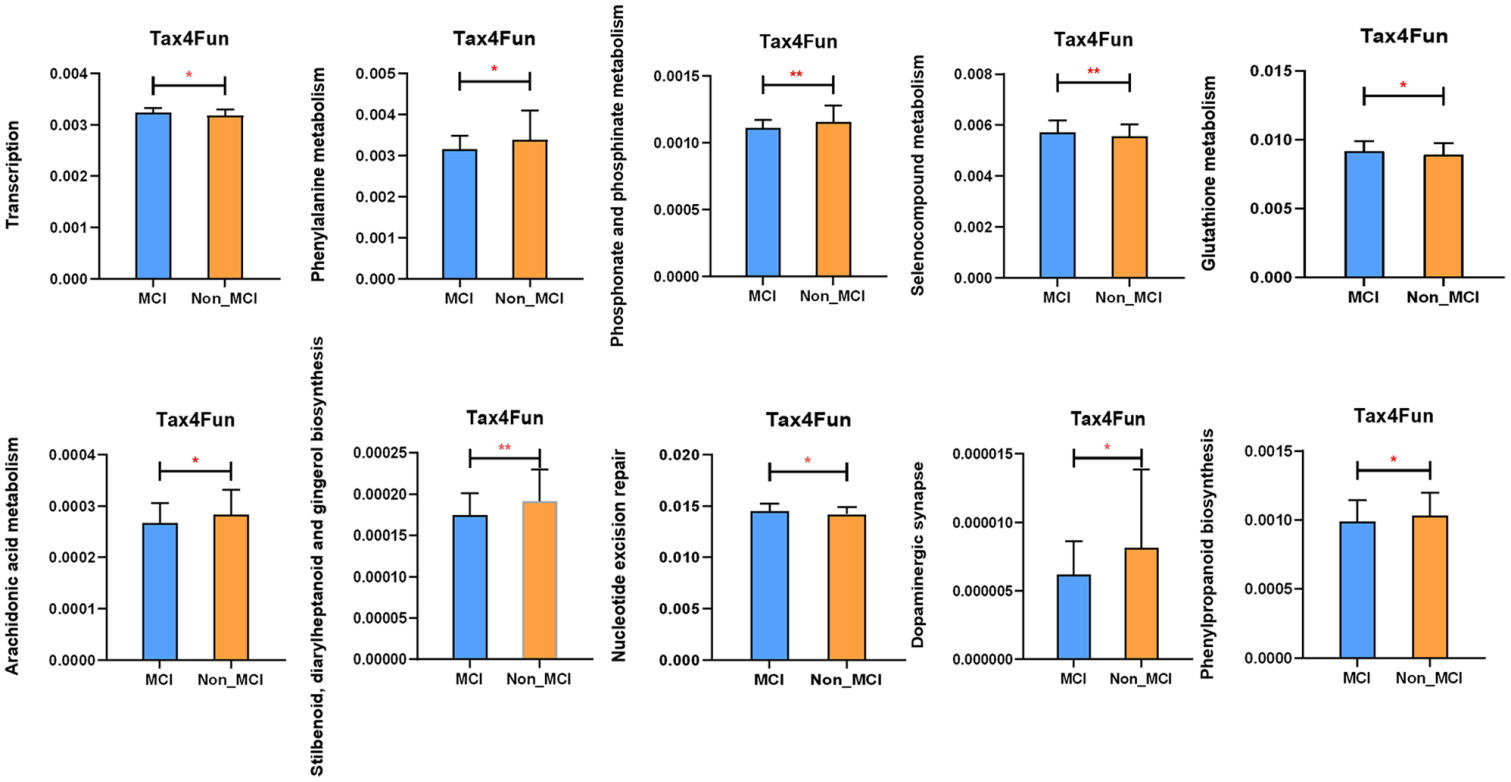
Tan4FUN function prediction. Tax4Fun function prediction analysis was used to compare the metabolic function of intestinal flora in two groups of stool samples. MCI: obesity with cognitive impairment group, n = 49; Non_MCI: obese but cognitively normal group, n = 55; *P < 0.05, **P < 0.01, ***P < 0.001

In this study, based on KEGG Level 1–3 abundance, the metabolic function of intestinal flora was predicted. Since the difference in Level 1 was not statistically significant, only 10 functional pathways with statistically different levels 2–3 were shown (*P* < 0.05). Compared to the Non_MCI group, the MCI group had a significant increase in the functional categories of Transcription, Selenocompound metabolism, Glutathione metabolism, and Nucleotide excision repair, while functional pathways such as Arachidonic acid metabolism decreased (Fig. 4).

## Discussion

Brain health is closely related to physical health, and bodily function is largely cognitively mediated (Kiiti Borges et al. 2019). MCI is considered a prodromal phase of AD and refers to the transitional period between normal aging and the stages of dementia (Petersen 2011). In recent years, dietary factors, especially fat intake (Ding et al. 2018), have been shown to be associated with hippocampal neurogenesis and cognitive development (Brown et al. 2019). According to the literature, the composition of fatty acids in the erythrocyte membrane is relatively stable, which can reflect the intake level of dietary fatty acids in the past few months (Fote et al. 2021). Erythrocyte membrane fatty acids can be used as biomarkers for a variety of metabolic diseases, including obesity, diabetes, metabolic syndrome, etc. (Svegliati-Baroni et al. 2019).

Higher intake of total monounsaturated fatty acid (MUFA) was positively correlated with cognitive function, the percentage of C24:1 in the erythrocyte fatty acid profile was lower in the MCI group and was statistically different between the two groups. Some studies have shown that diets rich in MUFA can reduce the risk of neurodegenerative diseases such as AD (Dohrmann et al. 2019), and enhance overall cognitive function (Okereke et al. 2012).

Our study also found that C18:3 n-3 in the erythrocyte membrane n-3 fatty acid profile was significantly different between the two groups. One study showed that supplementation of n-3 PUFA to obese patients revealed a significant reduction in the amount and percentage of abdominal fat in obese individuals and an increase in MoCA over time (Salman et al. 2022). In a prospective cohort study by Liu found that α-linolenic acid (ALA) intake was negatively associated with weight gain (Liu et al. 2018). The results of Nooyens’ cohort study suggest that a higher intake of n-3 polyunsaturated fatty acids (especially ALA) slows the decline in overall cognitive function and memory. (Nooyens et al. 2018). In turn, our findings suggest that higher levels of C18:3 n-3 in erythrocyte membranes may have a beneficial effect on cognition in obese patients.

Studies have shown that higher n-6 PUFA intake is inversely associated with cognitive function including language skills, delayed recall ability, and memory capacity (Shen et al. 2022). Several studies have shown that patients with cognitive impairment have higher levels of n-6 PUFA intake and that n-6 PUFA intake plays a role in contributing to the development of cognitive impairment in obese patients (Bigornia et al. 2018). In the previous study, a higher C20:4 n-6 was associated with an increased risk of dementia (Samieri et al. 2008). Our results are consistent with previous results. This may be due to the fact that n-6 PUFA levels in erythrocytes are associated with brain structure and amyloid plaques (Hooper et al. 2017). In addition, Heude et al.’s four-year, cohort study of 246 older adults (Heude et al. 2003) showed that older subjects with high levels of n-6 PUFA and low levels of n-3 PUFA in erythrocyte membranes were most likely to experience cognitive decline. Moreover, a study of 84 patients also found that the n-6/n-3 ratio was higher in AD, MCI and other dementia diseases (Conquer et al. 2000). Therefore, the ratio of dietary n-6/n-3 polyunsaturated fatty acid intake has a potential impact on cognitive decline (de Lorgeril et al. 1998). Our data indicate that an increase in the n-6/n-3 ratio may be associated with the development of cognitive impairment in obese patients.

In addition, in our study, the increased n-6/n-3 ratio of erythrocyte membrane in MCI group was mainly caused by higher n-6 PUFA in MCI group, especially higher levels of C18:2 n-6 and C20:4 n-6 in the lipid profile of n-6 PUFA, so higher intake of C18:2 n-6 and C20:4 n-6 may contribute to the development of cognitive impairment in obese patients. This may be due to the involvement of arachidonic acid in the production of eicosanoid and the inflammatory response of the body (Janssen and Kiliaan 2014). Studies have shown that high intake of linoleic acid can increase oxidative stress in the body, which can cause cognitive impairment (Corrêa Leite et al. 2001). Our results also showed not only higher levels of n-6 PUFA in erythrocyte membranes but also higher levels of inflammatory factors such as IL-3 and IL-12 in the blood of patients in the MCI group, which is consistent with previous studies suggesting that n-6 PUFA may reduce cognitive function by affecting the inflammatory response in humans.

Dietary n-6 PUFA may also alter miRNA expression profile (Zheng et al. 2015). Functional analysis of these changes in miRNA expression profiles suggests that dietary PUFA are associated with the maintenance of immune homeostasis through miRNA expression (Zanoaga et al. 2018). In vitro and in vivo studies have demonstrated the essential participation of some of these RNAs in the production and regulation of several proinflammatory cytokines (Huang et al. 2014). Zhang et al. (Zhang et al. 2021) noted a downregulation of miR-107 in a study of Chinese middle-aged and older populations, which was associated with an elevated risk of MCI. It has also been shown that miR-107 levels are found to be reduced in the plasma of patients with early MCI disease stages. This reduction correlates with disease severity and may effectively differentiate between AD and MCI patients. Additionally, the overexpression of miR-142 in the hippocampus or plasma from AD and MCI patients was confirmed through a meta-analysis that included 18 studies with 1027 participants (Shi et al. 2020). In the present study, it was also suggested that miR-107 levels decreased and miR-142 levels increased in the MCI group, though there was no statistical difference, which may be due to the small sample size or the fact that the MCI patients in this study were in the early stages of MCI and the disease was not yet too severe. Our results suggest that alterations in miR-107 and miR-142 levels may be associated with the onset of cognitive impairment in obese patients.

There is no doubt that obesity can lead to an imbalance in the body’s intestinal flora (Liu et al. 2021a). A recent animal study showed that a high n-6/n-3 ratio diet exacerbated intestinal flora dysbiosis in obese rats, suggesting that consuming excessive n-6 PUFA or deficient n-3 PUFA can lead to intestinal flora disorders in humans (Lee et al. 2019). Dysbiosis of gut microbes can facilitate or reduce the regulation of several neurochemical and neurometabolic pathways through complex gut-brain axis interactions (Junges et al. 2018), and can alter the permeability of the blood-brain barrier and its protective function (Spadoni et al. 2017), leading to the development of cognitive impairment. Several studies have shown that patients with cognitive impairment have a higher proportion of *Bacteroidota* in their stools compared to the cognitively normal group (Saji et al. 2019). In addition, patients with higher *Bacteroides* are more likely to experience cortical and hippocampal atrophy. Multivariate logistic regression analysis showed that higher *Bacteroidota* abundance was associated with MCI (Saji et al. 2019). Animal studies have shown that mice with AD have a decreased percentage of *Proteobacteria* and *Actinobacteria* in their intestines (Asadbegi et al. 2018). These results suggest that elevated abundance of *Bacteroidota* may have a contributory role in the development of mild cognitive impairment.

Our study analyzed the differences in intestinal flora species between the two groups, as well as their correlation with cognitive function scores and erythrocyte membrane fatty acids. Population and animal studies have shown that the relative abundance of *Clostridiaceae* is negatively correlated with cognitive function (Sun et al. 2019). Animal studies showed that the relative abundance of *Adlercreutzia* was high in AD mice (Wang et al. 2022). The results suggest a potential role for *Clostridiaceae and Adlercreutzia* in cognitive impairment. *norank_f_Lachnospiracea* is a member of the *Lachnospiraceae* family. Previous studies have shown that the *Lachnospiraceae* family is positively correlated with amyloid β-Protein 42 (Aβ42) (Nagpal et al. 2019). The occurrence of cognitive impairment in obese patients may be associated with increased abundance of *Lachnospiraceae* including *norank_Lachnospiracea*. Wang et al. (Wang et al. 2016) reported that IL-1β and tumor necrosis factor-α (TNF-α) transcript levels were positively correlated with *Clostridium_sensu_stricto_1* enrichment in the sheep colon. Another interesting and novel genus we found was *Coriobacteriales_Incertae_Sedis*, which was not only negatively correlated with cognitive scores in this study, but also positively correlated with erythrocyte membrane C20:4 n-6, n-6 PUFA. The results suggest that n-6 PUFA and *Coriobacteriales_Incertae_Sedis* play a role in the development of mild impairment in obese individuals.

The *Acidaminococcaceae* have been shown to produce propionic acid (Gallier et al. 2020), Nagpal et al. (Nagpal et al. 2019) studies showed that propionic acid in the cerebrospinal fluid of patients with mild cognitive impairment was negatively correlated with Aβ42, that is, the higher the abundance of *Acidaminococcaceae*, the better the cognitive performance. This is consistent with our findings.

Many potential functions of the gut microbiota affect systemic metabolism and may play a key role in the comorbid cognitive dysfunction of obese patients. In the functional category, we observed that some functional pathways were increased in the MCI group compared to the Non_MCI group, including Transcription, Selenocompound metabolism, Glutathione metabolism, and Nucleotide excision repair Many of these pathways may be related to cognition (Pulikkan et al. 2019; Wang et al. 2019). We therefore hypothesized that structural changes in the intestinal flora community could potentially affect host metabolism and thus cognitive function.

There are some limitations to our study. First, it is a cross-sectional design, so it cannot provide any evidence of causal conclusions. Therefore, validation needs to be carried out in clinical trials. Also, this study used questionnaires, and access to information depended on participants’ memory, which could lead to recall bias. As a result of the pandemic, a small percentage of participants may consciously change their eating habits, such as eating less seafood than before, resulting in a decrease in dietary intake of polyunsaturated fatty acids. Our study sample was relatively small. Since our participants are from the Beijing metropolitan area, the results are not necessarily representative of Beijing as a whole. The 16 S rRNA high-throughput sequencing samples are derived from PCR amplification products, and the “universal primers” used in the PCR process do not amplify all microorganisms equally, leading to a certain degree of distortion in the analysis results. As shown in supplementary material,Tax4Fun was based on the SILVA database and KEGG database for gene function prediction, and although the database has bacterial, fungal and archaeal rRNA gene sequences, the database has just been established and is not yet complete. Microbial taxa that are novel or uncharacterized might not have available reference genomes, which could lead to incomplete or biased functional predictions.

In the present study, we found that higher levels of n-6 PUFA in erythrocyte membranes may trigger inflammatory responses in body and may affect the expression of miRNA-142 and miRNA-107. High levels of n-6/n-3 polyunsaturated fatty acid ratio in the erythrocyte membrane may also lead to increased abundance of *Coriobacteriales_Incertae_Sedis* in the gut microbes of obese patients, which in turn may affect cognitive function.

### Electronic supplementary material

Below is the link to the electronic supplementary material.

Bioinformatics analysis

Supplementary figure

## Data Availability

The datasets generated during and/or analysed during the current study are not publicly available due to the patient privacy concerns, but are available from the corresponding author on reasonable request.
