# Supplementary material for: Association between erythrocyte membrane fatty acids and gut bacteria in obesity-related cognitive dysfunction
Source: AMB Express. 2023 Dec 20;13:148. doi: 10.1186/s13568-023-01655-3 (PMC10733235; doi:10.1186/s13568-023-01655-3)
Supplement: Supplementary file 1 — Bioinformatics analysis [file 13568_2023_1655_MOESM1_ESM.pdf]

Association between erythrocyte membrane fatty acids and gut bacteria in obesity-related cognitive dysfunction

Tong Zhao[1], Hongying Huang[1], Jinchen Li[1], Jingyi Shen[1], Cui Zhou[1], Rong Xiao[1] and Weiwei Ma[1*]

[1] School of Public Health, Beijing Key Laboratory of Environmental Toxicology, Capital Medical University, Beijing, 100069, People's Republic of China.

[*] Corresponding author: Weiwei Ma, School of Public Health, Beijing Key Laboratory of Environmental Toxicology, Capital Medical University, Beijing, 100069, People's Republic of China, Tel +86-10-83911651, Fax +86-10-83911651, Email weiweima@ccmu.edu.cn.

Bioinformatics analysis

All sequences were OTU divided according to different similarity levels. Extract non-repetitive sequences from optimized sequences, which was convenient to reduce the amount of redundant computation (Software platform: Uparse version 7.0.1090 http://drive5.com/uparse/) in the intermediate process of analysis; Remove of single sequences without duplicates (http://drive5.com/usearch/manual/singletons.html); The non-repetitive sequences (excluding single sequences) were OTU clustered according to 97% similarity, and the chimeras were removed during the clustering process to obtain the representative sequence of OTU. All optimized sequences were used as OTU representative sequences, and sequences with similarity of 97% or more to the representative sequences were selected to generate OTU tables. The composition of community species in each sample was calculated according to different taxonomic levels (domain, boundary, phylum, class, order, family, genus, species). Based on the results of OTU and its clustering analysis and the information analyzed, the structural composition of the intestinal flora could be analyzed at the level of each genus. The following intestinal flora data were obtained from the Meguiar's Cloud Platform (Meiji Biomedical Technology Co, Shanghai, China)(https://cloud.majorbio.com/page/project/overview.html).

Alpha diversity analysis was conducted on the intestinal flora of MCI and Non_MCI to understand the difference in richness, diversity and coverage between the two groups of intestinal flora, and the main alpha diversity indicators included Shannon, Simpson, Chao, Ace and Coverage index. Venn diagrams could be used to count the number of species (e.g., OTUs) common and unique to the MCI and Non_MCI groups of samples, and could provide a more visual representation of the similarity and overlap of species (e.g., OTUs) composition of environmental samples. (Software: R language version 3.3.1). The Rarefaction curve was constructed using the Coverage diversity index of each sample at different

sequencing depths to reflect the microbial diversity of each sample at different sequencing quantities. (Software: R language tools). PCoA analysis (Principal co-ordinates analysis), or principal coordinates analysis, was a non-constrained data dimensionality reduction analysis method used to study the similarity or difference in the composition of two sample communities of samples. (Software: R language version 3.3.1). PLS-DA analysis was used to analyze the similarity between groups for the grouped samples. PLS-DA (Partial Least Squares Discriminant Analysis), or partial least squares discriminant analysis, was a discriminant analysis method in multivariate data analysis techniques, often used to deal with classification and discriminant problems. (Software: R language version 3.3.1 plsda analysis and graphing in the mixOmics package). LEfSe analysis was used to distinguish biological taxa between two groups of samples. Linear discriminant analysis (LDA) was performed on samples according to different grouping conditions based on taxonomic composition to identify groups or species that had a significant differential impact on sample delimitation. (Software: http://huttenhower.sph.harvard.edu/galaxy/root?tool_id=lefse_upload). Tax4Fun function prediction, the 16S taxonomic spectrum based on Silva database was transformed into the taxonomic spectrum of prokaryotes in KEGG database. Based on the information from the KEGG database, KO, Pathway, and EC information could be obtained, and the abundance of each functional class could be calculated based on OTU abundance. After obtaining the functional abundance, spss v26.0 was used to compare the functional abundance of the two groups and Graphpad Prism was used for graphing.
