# Supplementary material for: Association between erythrocyte membrane fatty acids and gut bacteria in obesity-related cognitive dysfunction
Source: AMB Express. 2023 Dec 20;13:148. doi: 10.1186/s13568-023-01655-3 (PMC10733235; doi:10.1186/s13568-023-01655-3)
Supplement: Supplementary file 2 — Supplementary figure [file 13568_2023_1655_MOESM2_ESM.pdf]

Association between erythrocyte membrane fatty acids and gut bacteria in obesity-related cognitive dysfunction

Tong Zhao[1], Hongying Huang[1], Jinchen Li[1], Jingyi Shen[1], Cui Zhou[1], Rong Xiao[1] and Weiwei Ma[1*]

[1] School of Public Health, Beijing Key Laboratory of Environmental Toxicology, Capital Medical University, Beijing, 100069, People's Republic of China.

[*] Corresponding author: Weiwei Ma, School of Public Health, Beijing Key Laboratory of Environmental Toxicology, Capital Medical University, Beijing, 100069, People's Republic of China, Tel +86-10-83911651, Fax +86-10-83911651, Email weiweima@ccmu.edu.cn.

Supplementary Fig S1 Correlation analysis of erythrocyte membrane fatty acids and plasma cytokines

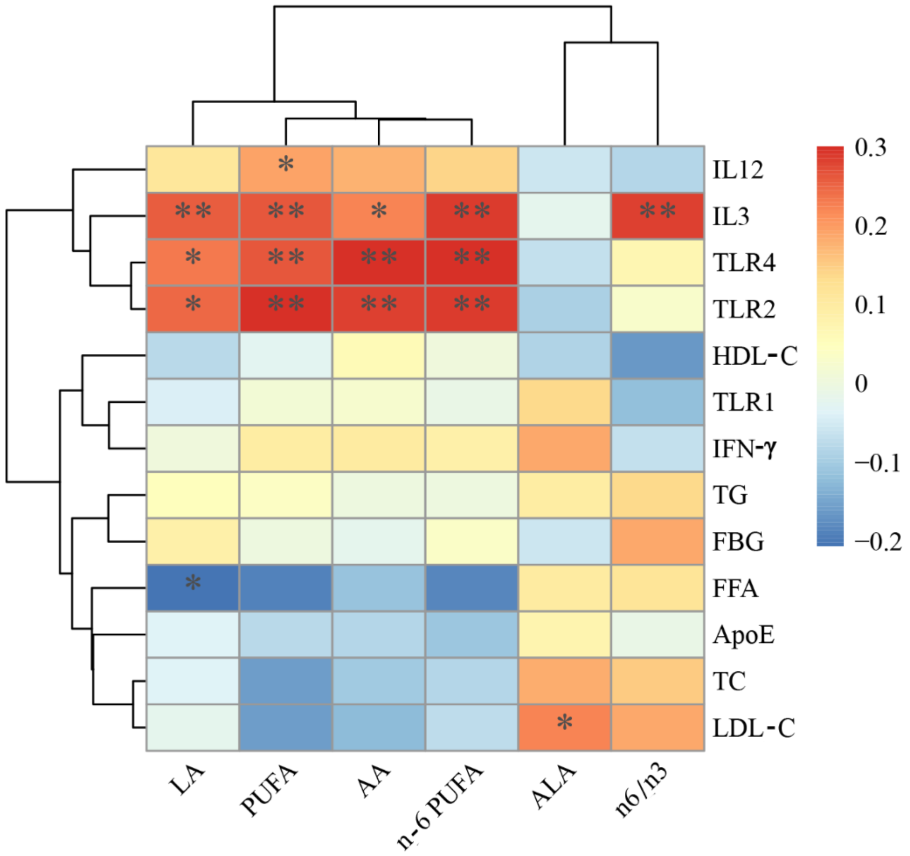

Legend Heat map of erythrocyte fatty acid and cytokine correlation. TC: total cholesterol; TG: total triglycerides; HDL-C: High-Density Lipoprotein Cholesterol; LDL-C: Low-Density Lipoprotein Cholesterol; FBG: fasting blood glucose; FFA: free fatty acids; ApoE: apolipoprotein E; TLR1: toll-like receptor 1; TLR2: toll-like receptor 2; TLR4: toll-like receptor 4; IL-3: interleukin-3; IL-12: interleukin 12; IFN-γ: interferon gamma; LA: Linoleate; PUFA: polyunsaturated fatty acid; n-6 PUFA: n-6 polyunsaturated fatty acid; n-6/n-3: Ratio of n-6 unsaturated fatty acids to n-3 unsaturated fatty acids; AA: Arachidonic acid; ALA: α-Linolenic acid. Red indicates positive correlation, blue negative correlation.

Supplementary Fig S2 Basic information of two groups of intestinal flora

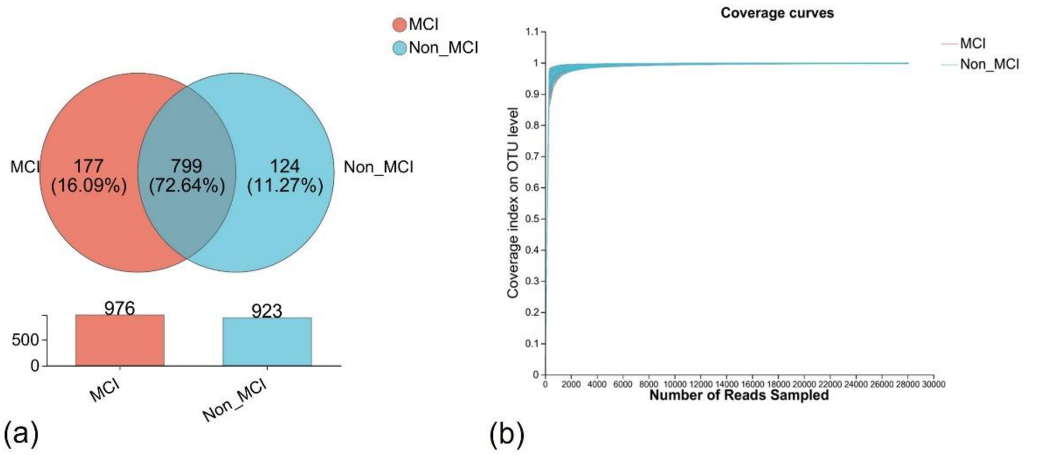

Legend (a) The common and unique species in the two groups of samples were compared, and the similarity and overlap of species composition in the samples were more intuitively expressed. (b) Select the OTU level with 97% similarity, use mothur to calculate the coverage index under random sampling, and use R language tools to make graphs. MCI: obesity with cognitive impairment group; Non_MCI: obese but cognitively normal group; OTU, operational taxonomic units.
